# Immune Dysregulation and Prognosis in Sepsis: Insights From a Posttranslational Perspective

**DOI:** 10.1155/humu/5503939

**Published:** 2025-07-08

**Authors:** Yifeng Ye, Qiaoling Chen, Dongjin Wu, Gaojie Yu, Jia'nan Liu, Yang Li, Zhihong Xu, Liang Lin

**Affiliations:** ^1^Department of Anesthesiology, The First Affiliated Hospital of Xiamen University, School of Medicine, Xiamen University, Xiamen, China; ^2^Xiamen Anesthesia Quality Control Center, Xiamen, China

**Keywords:** immune response, palmitoylation, prognostic biomarker, sepsis, ZDHHC19

## Abstract

Sepsis is a life-threatening condition triggered by infection, resulting in widespread inflammation, immune dysfunction, and multiorgan failure. Despite advances in medical science, early detection, treatment, and prognosis remain significant challenges. In this study, we investigated the expression and functional role of ZDHHC19, a palmitoyltransferase enzyme, in sepsis. Our results demonstrate that ZDHHC19 is significantly upregulated in neutrophils during sepsis and correlates with inflammatory pathways critical to disease progression. High ZDHHC19 expression was associated with increased activation of immune-inflammatory processes such as collagen metabolism, myeloid cell activation, and cell adhesion, while suppressing antigen presentation. Additionally, ZDHHC19 expression was linked to poor patient prognosis, with higher levels correlating with increased mortality and sepsis shock. Using advanced computational tools, including XGBoost for machine learning-based core gene selection and CellChat for cell communication analysis, we identified key regulatory networks modulated by ZDHHC19, revealing its pivotal role in immune dysregulation. These findings suggest that ZDHHC19 may serve as a potential biomarker for the diagnosis and prognosis of sepsis and open new avenues for targeted therapeutic interventions aimed at modulating immune responses in this condition.

## 1. Introduction

Sepsis is a life-threatening condition triggered by infection, resulting in widespread inflammation, immune dysfunction, and multiorgan failure [1–3]. It remains one of the leading causes of death, particularly in critically ill patients [4]. This complex, acute response to infection involves a dysregulated immune response that leads to systemic inflammation, often accompanied by septic shock, organ dysfunction, and death [5, 6]. Despite advances in medical science, the early detection, treatment, and prognosis of sepsis remain significant challenges [7]. The immune system, initially mobilized to combat the infection, may become overwhelmed, causing excessive inflammation, tissue damage, and even immune suppression [8, 9]. The progression of sepsis involves interactions between pathogens, the immune system, and host cells, making it difficult to diagnose and treat [10]. Early diagnosis is crucial, as delayed or inadequate treatment significantly increases mortality rates [11, 12]. Although the management of sepsis primarily includes antimicrobial therapy and supportive care to address organ dysfunction, therapeutic strategies are still limited and their efficacy relies on timely and accurate identification of sepsis onset [13]. Recent research is focused on identifying biomarkers and molecular signatures to improve early detection, treatment strategies, and prognostication.

Recent advances in high-throughput technologies, such as single-cell RNA sequencing, have provided unprecedented insights into the molecular landscape of diseases [14, 15]. These technologies enable the analysis of individual cells at a molecular level, providing a detailed view of the immune cell composition, gene expression patterns, and cellular interactions that occur during sepsis [16, 17]. The immune system's response to sepsis is complex and involves a diverse array of cell types, including neutrophils, monocytes, T cells, B cells, and macrophages, all of which play distinct roles in the pathogenesis of sepsis [18–20]. Understanding the dynamics of these cells and their molecular signatures could potentially lead to the identification of biomarkers that not only provide insights into the disease mechanisms but also enable early diagnosis and prediction of disease outcomes.

ZDHHC19, a member of the DHHC (Asp-His-His-Cys) family of palmitoyltransferases, is involved in the posttranslational modification of proteins through palmitoylation, which regulates various cellular functions, including protein trafficking, signal transduction, and membrane interactions [21, 22]. Palmitoylation is a reversible lipid modification that attaches a palmitate group to cysteine residues on target proteins, influencing their localization and function [23, 24]. Palmitoylation is known to play critical roles in immune cell activation, signaling, and responses to inflammatory stimuli [25, 26]. Therefore, understanding the role of ZDHHC19 and its associated palmitoylation activity in immune cells during sepsis could provide valuable insights into the pathophysiology of sepsis.

ZDHHC19's role in immune cell regulation has attracted considerable attention, especially in the context of inflammatory diseases [27]. Previous studies have shown that ZDHHC19 influences immune cell signaling and function, but its role in sepsis, particularly its impact on immune cell subsets and inflammatory pathways, is not yet fully understood. Given the importance of immune cells in sepsis, especially neutrophils, macrophages, and T cells, studying the expression and function of ZDHHC19 in these cells may offer novel therapeutic targets for sepsis management. In addition to its potential role in immune modulation, ZDHHC19 has been implicated in various cancers, where it is thought to regulate tumor progression and immune cell interactions within the tumor microenvironment [28]. The parallels between cancer and sepsis, particularly the dysregulated immune response and inflammation, highlight the relevance of investigating ZDHHC19 in the context of sepsis. Moreover, understanding the molecular pathways and cellular networks regulated by ZDHHC19 could contribute to the development of targeted therapies that modulate immune responses in sepsis, similar to cancer immunotherapies that aim to restore immune system function.

Although extensive efforts have been made to characterize the immune dysregulation underlying sepsis, much of the existing literature has focused on broad immune responses or canonical pathways, with limited emphasis on posttranslational modifications such as palmitoylation and their role in immune cell function during sepsis. Moreover, while several DHHC family members have been studied in cancer and neurological disorders, their specific contributions to sepsis pathophysiology remain poorly understood, and ZDHHC19, in particular, has not been systematically explored in this context. The lack of comprehensive data on how ZDHHC19 modulates innate immune responses, especially in neutrophils—key effector cells in sepsis—represents a significant gap in current knowledge. This study addresses this gap by integrating single-cell transcriptomics, machine learning, and cell communication analysis to investigate the expression patterns, immune associations, and prognostic relevance of ZDHHC19 in sepsis. By focusing on its role in neutrophil activation, immune signaling, and disease severity, our findings provide new insights into the molecular mechanisms driving immune dysregulation in sepsis and propose ZDHHC19 as a potential mediator of disease progression.

## 2. Methods

### 2.1. Data Collection

All data used in this study were obtained from the Gene Expression Omnibus (GEO) database (https://www.ncbi.nlm.nih.gov/geo/). Dataset selection was based on the following inclusion criteria: (1) relevance to human sepsis with both sepsis and control samples, (2) adequate sample size (≥ 10 samples per group) to support reliable statistical analysis, (3) availability of raw or normalized gene expression matrices, and (4) presence of essential clinical metadata, such as survival information. Datasets were excluded if they lacked clinical annotations, had low sequencing quality, or were not related to immune or transcriptomic profiling in sepsis. For single-cell RNA-sequencing analysis, we selected GSE167363 and GSE175453, as both contain high-quality, well-annotated immune cell profiles from sepsis and control samples, allowing for a detailed investigation of immune heterogeneity across 21 cases. To mitigate batch effects between datasets, we employed the Harmony algorithm during integration and applied standard preprocessing workflows, including mitochondrial filtering, doublet removal, and normalization using the SCTransform method.

For bulk RNA analysis, four independent datasets—GSE13904 (18 control, 158 sepsis), GSE9960 (15 control, 30 sepsis), GSE26440 (32 control, 98 sepsis), and GSE32707 (34 control, 89 sepsis)—were used to identify core palmitoylation genes through machine learning. Specifically, XGBoost was employed to evaluate gene importance across cohorts. To assess the prognostic relevance of candidate genes, we further analyzed three datasets with survival outcome data: GSE26378 (103 sepsis patients), GSE26440 (116 patients), and GSE95233 (102 patients). The use of multiple independent cohorts for both discovery and validation phases strengthens the reliability and generalizability of our findings.

### 2.2. Data Processing

For single-cell data analysis, Seurat Version 4.2.2 was used. Specifically, after reading in each dataset, quality control was performed by removing cells with nFeature_RNA < 200 or > 5000, or with mitochondrial content exceeding 10% [29]. While certain immune cell types, such as neutrophils, are known to exhibit slightly higher mitochondrial RNA proportions, previous single-cell transcriptomic studies have shown that a 10% threshold remains appropriate for preserving cell viability and minimizing low-quality or stressed cells, including within neutrophil populations [30]. The SCTransform function was used for data standardization and normalization. The first 15 principal components were selected for dimensionality reduction and clustering, with the RunUMAP function used to compute the dimensionality reduction results. The DoubletFinder package was used to remove doublets. The Harmony algorithm was employed to remove batch effects between different datasets and samples. Cell annotation was performed using SingleR and previous literature. The FindAllMarkers function was used to analyze marker genes for each cell type, and the ggplot2 package was used for visualizing the UMAP results. For bulk RNA data, the limma package was used for data standardization and normalization. The Survival and survminar packages were used for survival curve analysis and plotting.

### 2.3. Ro/e Analysis

Ro/e is the ratio of the observed cell quantity to the expected cell quantity for each cell cluster in different tissue types. The expected cell number for each cell cluster and tissue combination was obtained using a chi-square test. Unlike the traditional chi-square test, which only indicates the difference between observed and expected values, the chi-square value defined by Ro/e can indicate whether a specific cell cluster is enriched or depleted in a particular tissue. For example, if Ro/*e* > 1, it suggests that the specific T cell cluster is more frequently observed in the tissue than expected, indicating enrichment. If Ro/*e* < 1, it suggests that the cell cluster is observed less frequently than expected, indicating depletion. Ro/*e* calculation allows for an effective quantification of cell cluster tissue preference.

### 2.4. Cell Communication

The “CellChat” R package (Version 1.5.0) was used to reveal the potential mechanisms of cell-to-cell communication at the single-cell level. The createCellChat function was used to build the CellChat object, and the aggregateNet function described the signals emitted from each cell type. The netVisual_circle function was used to display the number and weight of cell-to-cell communications, and the netAnalysis_computeCentrality function inferred the input and output weights for specific signaling pathways.

### 2.5. Gene Set Enrichment Analysis

To increase the reliability of the scoring, three scoring methods were used, including AUCell, JASMNE, and ssGSEA. The scoring was based on the irGSEA package, and the irGSEA.score function was used to calculate scores for each sample using three algorithms on the normalized matrix. The irGSEA.integrate function was applied to compute the total score for different cell types, and the final visualization was performed using the irGSEA.halfvlnplot function.

### 2.6. Differential Expression and Enrichment Analysis

Differential expression analysis was performed using the limma package for different groups, with logFC absolute values greater than 1 and adjusted *p* values less than 0.05 considered significant. Enrichment analysis of all differentially expressed genes was carried out by first sorting genes according to their logFC values, followed by GSEA and clusterProfiler package enrichment analysis for three gene sets, including GOBP, KEGG, and REACTOME. The filtering criterion was *p* value < 0.05. The ggplot2 package was used for visualization.

### 2.7. Machine Learning and Core Gene Selection

To identify core palmitoylation genes associated with sepsis, the classic machine learning method XGBoost was employed. XGBoost, short for eXtreme Gradient Boosting, is an effective implementation of gradient boosting commonly used in machine learning analysis. The xgboost package was used for the machine learning analysis, and the DALEX and breakDown packages were used for result interpretation. Multiple datasets were used to compute the partial dependence of the palmitoylation genes, with higher values indicating more important genes. The pROC package was used to plot ROC curves.

### 2.8. Immune-Related Analysis

GSVA and GSEABase packages were used to calculate the relative enrichment scores for 29 immune cell types and immune processes. The algorithm was based on the ssGSEA strategy, and the expression matrix of sepsis core genes was correlated with immune cells and immune processes, with correlation matrices plotted. The expression of chemokine family and HLA family molecules was analyzed for correlation with sepsis palmitoylation core genes, and the ggplot2 package was used to plot correlation heatmaps.

### 2.9. Pan-Cancer Analysis

Pan-cancer analysis was performed using the TCGAplot package, with pan_boxplot and pan_paired_boxplot functions used for expression box plots and paired box plots. The gene_immucell_heatmap function was used to analyze the correlation of immune cell abundance with ZDHHC19, and the gene_immunescore_heatmap function was used for pan-cancer immune score correlation analysis.

## 3. Results

### 3.1. Sepsis Cell Types and Molecular Change Analysis

**Figure 1:**
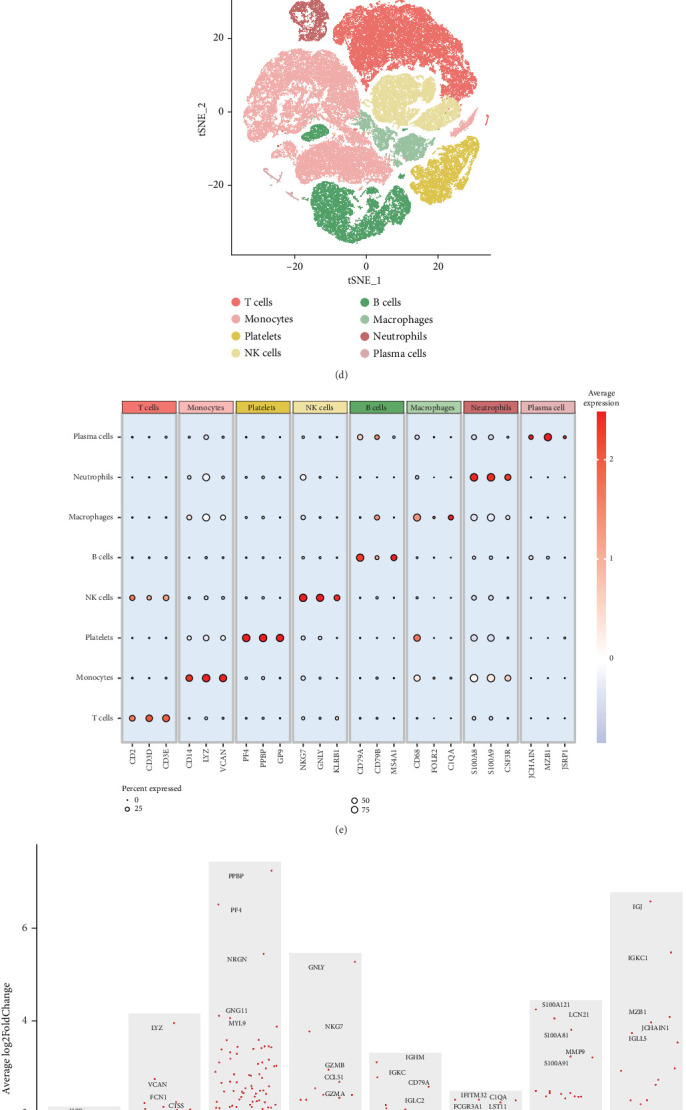
Cell types and molecular changes in sepsis. (a, b) UMAP plots showing the integration of two sepsis datasets (21 samples in total) after removing batch effects using the Harmony algorithm. A total of 86,329 cells were retained, including 33,109 control cells and 53,220 sepsis cells. (c) Clustering of cells: tSNE plot of 24 clusters (0–23) obtained from clustering the integrated dataset after adjusting the resolution. (d–f) Annotation of clusters based on highly expressed genes for each cluster, identifying eight distinct cell populations: T cells (CD2, CD3D, and CD3E), monocytes (CD14, LYZ, and VCAN), platelets (PF4, PPBP, and GP9), NK cells (NKG7, GNLY, and KLRB1), B cells (CD79A, CD79B, and MS4A1), macrophages (CD68, FOLR2, and C1QA), neutrophils (S100A8, S100A9, and CSF3R), and plasma cells (JCHAIN, MZB1, and JSRP1). (g) Enrichment analysis using the Ro/e algorithm, highlighting the predominant cell types in control and sepsis tissues, with neutrophils being significantly enriched in sepsis. (h) Cell–cell communication analysis showing the signaling output from monocytes, B cells, plasma cells, and macrophages, suggesting their pivotal role in the inflammatory microenvironment during sepsis. (i) Activation of key signaling pathways, including MIF and ANNEXIN, in monocytes, based on both input and output signaling analysis.

To provide a comprehensive interpretation of the cell types and molecular changes in sepsis, we systematically integrated two sepsis datasets and removed batch effects using the Harmony algorithm. The UMAP plot demonstrates that the samples were well integrated, with a total of 21 samples (Figure 1a,b). After quality control, 86,329 cells were retained, consisting of 33,109 control cells and 53,220 sepsis cells. By adjusting the resolution, we clustered all cells into 24 clusters (0–23) (Figure 1c). Based on the highly expressed genes of each cluster, we annotated them into eight distinct cell populations. The specific markers used for annotation are as follows: T cells (CD2, CD3D, and CD3E), monocytes (CD14, LYZ, and VCAN), platelets (PF4, PPBP, and GP9), NK cells (NKG7, GNLY, and KLRB1), B cells (CD79A, CD79B, and MS4A1), macrophages (CD68, FOLR2, and C1QA), neutrophils (S100A8, S100A9, and CSF3R), and plasma cells (JCHAIN, MZB1, and JSRP1) (Figures 1d, 1e, and 1f).

### 3.2. Cell Types Critical in Sepsis Development

To identify key cell types crucial for sepsis development, we employed the Ro/*e* algorithm, a commonly used method for evaluating tissue preference, as reported in several studies. The analysis revealed that monocytes and T cells were predominantly enriched in control tissues, while in sepsis, most cell types were enriched, particularly neutrophils. This suggests that neutrophils may play a significant role in the onset and progression of sepsis (Figure 1g). Cell communication analysis further showed that monocytes, B cells, plasma cells, and macrophages exert strong signaling outputs to other peripheral cells, indicating their pivotal role in the inflammatory microenvironment during sepsis (Figure 1h). In-depth signaling pathway analysis revealed that MIF, ANNEXIN, and other pathways were significantly activated in monocytes, both in terms of input and output (Figure 1i).

### 3.3. Palmitoylation in Sepsis: A Key Pathophysiological Process

**Figure 2:**
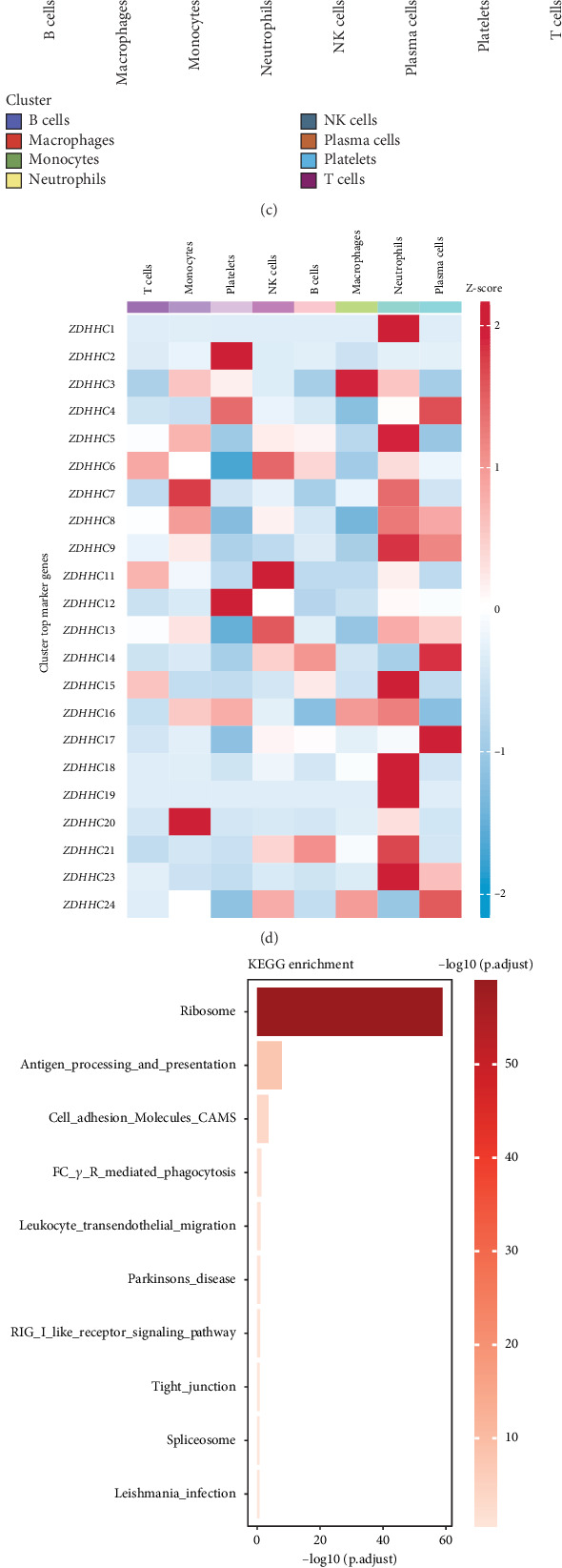
Palmitoylation in sepsis: a key pathophysiological process. (a–c) Palmitoylation scores across various cell types in sepsis, with neutrophils exhibiting significantly higher palmitoylation scores compared to other cell types. (d) Heatmap showing the expression of palmitoylation-related genes (e.g., ZDHHC1 and ZDHHC9) across different cell types, with neutrophils displaying the highest expression of these genes. (e–g) Enrichment analysis revealing that neutrophils are primarily involved in defense responses, recruitment of innate immune cells, and activation of pattern recognition receptors in sepsis.

Palmitoylation is a critical and highly studied pathophysiological process. To assess which cells in sepsis were strongly correlated with this process, we employed three scoring algorithms. The results consistently showed that neutrophils exhibited significantly higher palmitoylation scores compared to other cell types (Figures 2a, 2b, and 2c). Most palmitoylation-related genes, such as ZDHHC1 and ZDHHC9, were highly expressed in neutrophils (Figure 2d). Enrichment analyses of neutrophils revealed that these cells were primarily involved in the upregulation of defense responses, recruitment of innate immune cells, and activation of pattern recognition receptors (Figures 2e, 2f, and 2g).

### 3.4. Subclassification of Neutrophils in Sepsis

**Figure 3:**
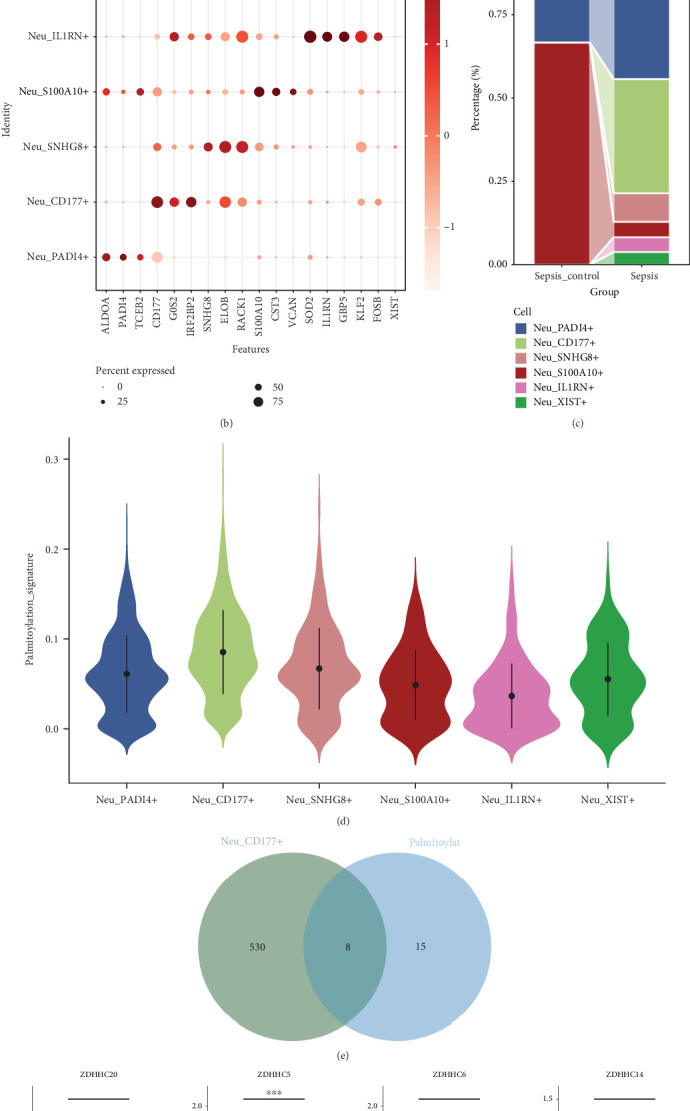
Subclassification of neutrophils in sepsis. (a, b) tSNE plot showing the subdivision of neutrophils in sepsis based on optimal resolution, with each subgroup expressing distinct markers such as PADI4, CD177, SNHG8, S100A10, IL1RN, and XIST. (c) Cell percentage graph illustrating an increased proportion of PADI4+, CD177+, and SNHG8+ neutrophils in sepsis tissues. (d) Violin plot comparing palmitoylation scores across neutrophil subgroups, with CD177+ neutrophils exhibiting the highest scores. (e) Differential expression analysis of palmitoylation-related genes in CD177+ neutrophils, identifying eight significantly differentially expressed genes. (f) Violin plots showing the upregulation of ZDHHC5, ZDHHC16, ZDHHC17, and ZDHHC19 in sepsis.

Further classification of neutrophils based on the optimal resolution revealed six subgroups, each highly expressing PADI4, CD177, SNHG8, S100A10, IL1RN, and XIST (Figure 3a,b). The cell percentage graph indicated that PADI4+ neutrophils, CD177+ neutrophils, and SNHG8+ neutrophils were significantly increased in sepsis tissues (Figure 3c). Among these, CD177+ neutrophils exhibited the highest palmitoylation scores (Figure 3d). A differential expression analysis of the palmitoylation-related genes in CD177+ neutrophils revealed eight palmitoylation-related genes with differential expression (Figure 3e). Further violin plot analysis of the expression of these eight genes in control and sepsis cells demonstrated that ZDHHC5, ZDHHC16, ZDHHC17, and ZDHHC19 were significantly upregulated in sepsis (Figure 3f).

### 3.5. Core Gene Identification Using XGBoost Machine Learning

**Figure 4:**
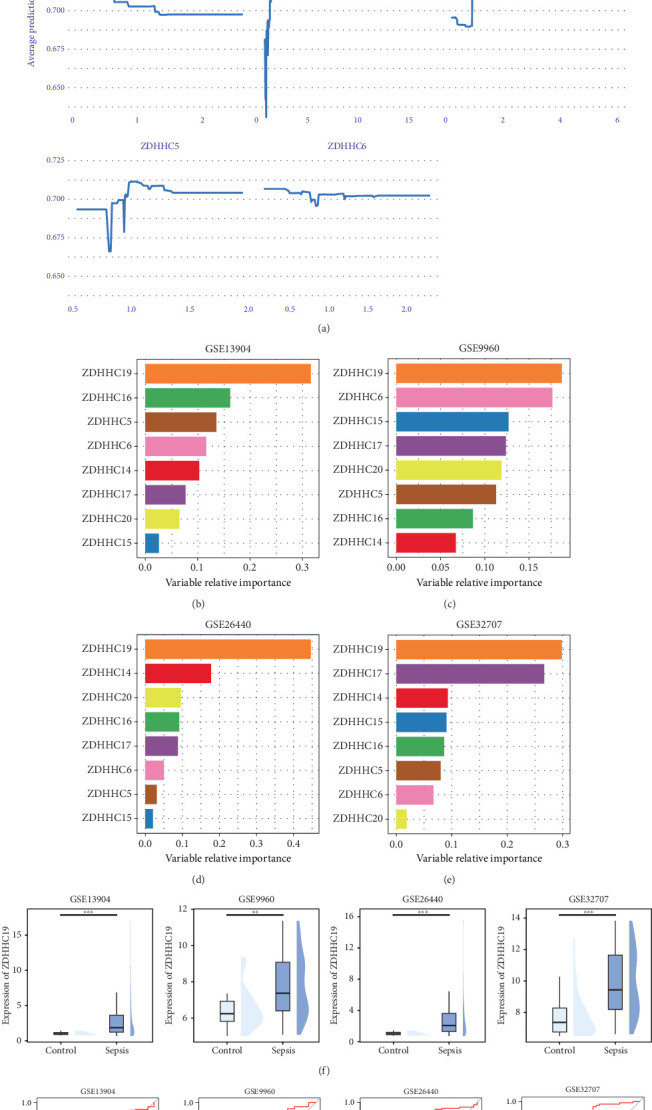
Core gene identification using XGBoost machine learning. (a) Partial dependence plots for the most important features (ZDHHC14, ZDHHC15, ZDHHC16, ZDHHC17, ZDHHC19, ZDHHC5, ZDHHC6, and ZDHHC20) selected using XGBoost machine learning, with ZDHHC19 showing the most significant change. (b–e) Feature importance ranking for eight differential palmitoylation-related genes across four sepsis datasets (GSE13904, GSE9960, GSE26440, and GSE32707), consistently placing ZDHHC19 at the highest rank. (f) Violin plots showing ZDHHC19 expression across four datasets, with significantly higher expression in sepsis patients compared to controls. (g) ROC curve analysis demonstrating the diagnostic performance of ZDHHC19 in sepsis, with AUC values ranging from 0.71 to 0.87 across four datasets, highlighting ZDHHC19 as a promising diagnostic marker for sepsis.

XGBoost machine learning was used for further core gene selection, with feature importance and dependency being crucial metrics for evaluating core genes. Based on the feature drop-out in the training set (GSE13904), ZDHHC19 showed the most significant change (Figure 4a). Feature importance scoring for the eight differential palmitoylation-related genes across four sepsis datasets consistently showed ZDHHC19 ranked highest (Figures 4b, 4c, 4d, and 4e). At the expression level, ZDHHC19 was found to be highly expressed in sepsis patients across all four datasets (Figure 4f). Furthermore, ZDHHC19 demonstrated excellent diagnostic performance, with an AUC above 0.7 in the ROC curve for all four datasets, with the largest AUC reaching 0.871, suggesting that ZDHHC19 could be a promising diagnostic molecular marker for sepsis (Figure 4g).

### 3.6. Expression of ZDHHC19 and Its Functional Enrichment

**Figure 5:**
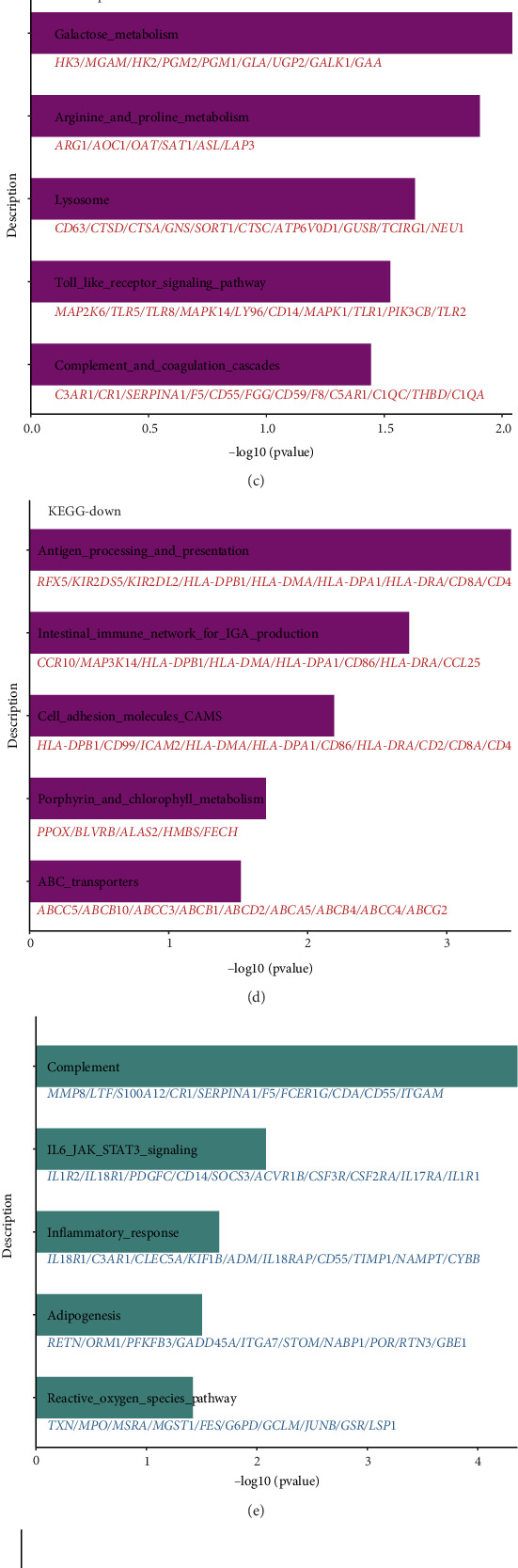
ZDHHC19 expression and gene enrichment analysis in sepsis. (a, b) Gene Ontology Biological Process (GOBP) enrichment analysis showing that high ZDHHC19 expression in sepsis patients significantly activates processes like collagen metabolism, myeloid cell activation, and cell adhesion, while low ZDHHC19 expression mainly activates processes related to antigen presentation. (c, d) Kyoto Encyclopedia of Genes and Genomes (KEGG) enrichment analysis revealing that high ZDHHC19 expression upregulates metabolic activity and pattern recognition receptor expression, while downregulating antigen presentation processes. (e, f) HALLMARK enrichment analysis indicating that high ZDHHC19 expression activates inflammatory responses and related pathways, while downregulating interferon signaling.

In the training set, patients were divided into high and low ZDHHC19 expression groups based on the median value of ZDHHC19 expression. Gene Ontology Biological Process (GOBP) enrichment analysis revealed that patients with high ZDHHC19 expression significantly activated processes such as collagen metabolism, myeloid cell activation, and cell adhesion. In contrast, patients with low ZDHHC19 expression mainly activated processes related to antigen presentation (Figure 5a,b). Kyoto Encyclopedia of Genes and Genomes (KEGG) enrichment analysis showed that high expression of ZDHHC19 upregulated metabolic activity and pattern recognition receptor expression, while downregulating processes related to antigen presentation (Figure 5c,d). Additionally, HALLMARK enrichment analysis also indicated that high ZDHHC19 expression activated inflammatory responses and related pathways, while downregulating interferon signaling (Figure 5e,f). Overall, ZDHHC19 may accelerate the inflammatory response by promoting the aggregation of immune-inflammatory cells, while simultaneously downregulating antibacterial responses, such as antigen presentation and interferon responses, thus contributing to the progression of sepsis.

### 3.7. ZDHHC19 and Sepsis Prognosis

**Figure 6:**
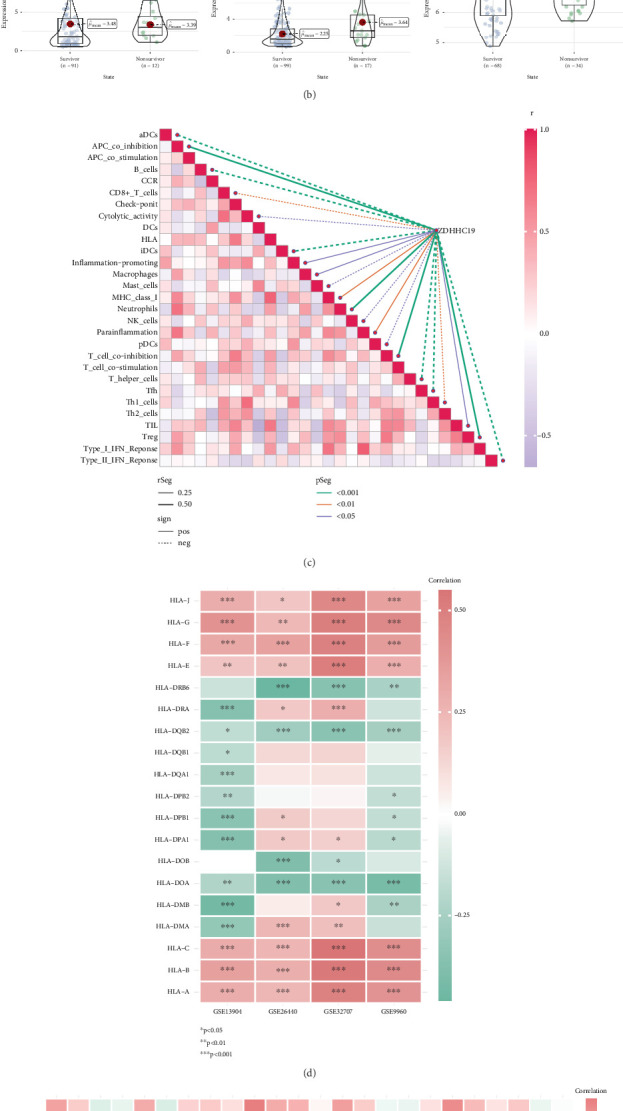
ZDHHC19 expression and sepsis prognosis. (a) Expression levels of ZDHHC19 in sepsis shock patients across three datasets, with high ZDHHC19 expression linked to the progression of sepsis. (b) Survival analysis showing that higher levels of ZDHHC19 correlate with increased mortality in sepsis patients. (c) ssGSEA analysis revealing that ZDHHC19 expression is negatively correlated with most immune cells (e.g., aDC cells, B cells, CD8+ T cells, iDC cells, and Th cells) but positively correlated with immunosuppressive cells such as Tregs. (d) ZDHHC19 expression shows a positive correlation with MHC Class I molecules but a negative correlation with MHC Class II molecules, suggesting a role in disease progression by modulating immune responses. (e) Chemokine analysis indicating that ZDHHC19 expression is positively correlated with various chemokines, supporting its proinflammatory role.

ZDHHC19 also holds prognostic significance for sepsis. ZDHHC19 was highly expressed in sepsis shock patients across three datasets, indicating that its high expression may contribute to disease progression (Figure 6a). Similarly, high expression of ZDHHC19 was associated with patient survival, with higher levels of ZDHHC19 correlating with increased mortality (Figure 6b). In terms of immunity, ssGSEA assessed 29 immune cell types and immune processes, revealing that ZDHHC19 expression was negatively correlated with most immune cells, including aDC cells, B cells, CD8+ T cells, iDC cells, and Th cells. However, it showed a significant positive correlation with certain immunosuppressive cells, such as regulatory T cells (Tregs) (Figure 6c). Regarding HLA family molecules, ZDHHC19 was positively correlated with MHC Class I molecules but negatively correlated with MHC Class II molecules. Given the importance of MHC II molecules in sepsis, this suggests that ZDHHC19 may promote disease progression (Figure 6d). Chemokine analysis showed that ZDHHC19 was generally positively correlated with chemokines, indicating its proinflammatory effect (Figure 6e).

### 3.8. ZDHHC19 Expression and Its Correlation With Neutrophils

**Figure 7:**
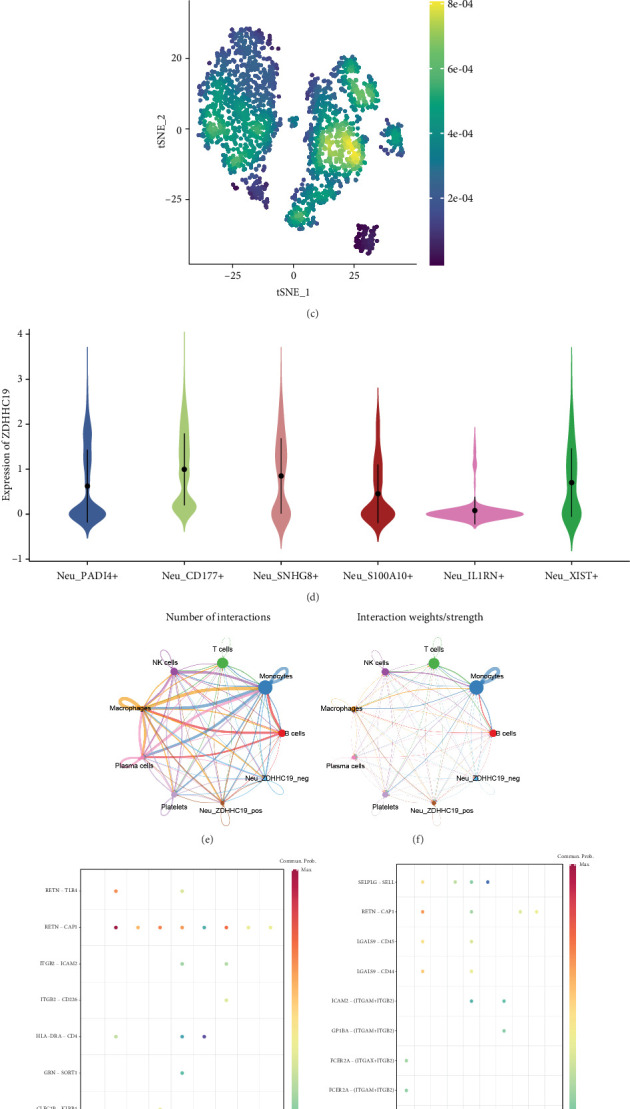
ZDHHC19 expression and its correlation with neutrophils. (a, b) ZDHHC19 expression levels in different cell types, with neutrophils showing the highest expression among all cell types. (c, d) ZDHHC19 expression in neutrophil subgroups, with CD177+ neutrophils exhibiting the highest expression levels, indicating specificity in these cells. (e–h) Cell communication analysis showing that ZDHHC19-positive neutrophils exhibit the greatest communication with macrophages. (i) ZDHHC19-positive neutrophils show significantly downregulated glucose and lipid metabolism, suggesting a close association with metabolic pathways.

Further analysis of gene and cell correlation revealed that ZDHHC19 was primarily expressed in neutrophils, with the highest expression observed in this cell type among all cells (Figure 7a,b). ZDHHC19 expression was also most concentrated in CD177+ neutrophils, and among all neutrophils, CD177+ neutrophils exhibited the highest expression of ZDHHC19, indicating high specificity for its expression in these cells (Figure 7c,d). Neutrophils were categorized into ZDHHC19-positive and -negative groups based on the presence or absence of ZDHHC19 expression. Cell communication analysis showed that ZDHHC19-positive neutrophils exhibited the greatest communication with macrophages, and receptor-ligand analysis indicated that the RETN-CAP1 receptor-ligand pair had the highest communication probability, interacting with monocytes, T cells, NK cells, macrophages, and others (Figures 7e, 7f, 7g, and 7h). In terms of metabolism, ZDHHC19-positive neutrophils exhibited significantly downregulated glucose and lipid metabolism, suggesting a strong association between ZDHHC19 expression and metabolic pathways (Figure 7g).

### 3.9. ZDHHC19 in Pan-Cancer Analysis

**Figure 8:**
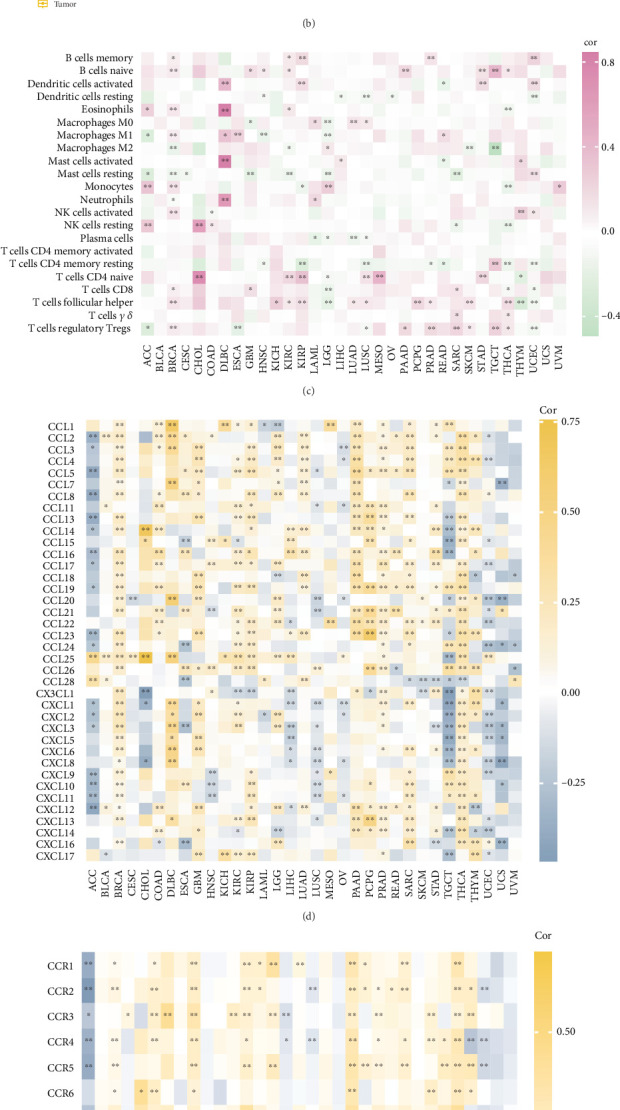
ZDHHC19 in pan-cancer analysis. (a, b) Box plots and paired box plots showing that ZDHHC19 is significantly upregulated in most cancer types, including bladder cancer and breast cancer, suggesting its role as a common oncogene. (c) Correlation analysis of ZDHHC19 expression and immune cells across different cancer types, revealing that ZDHHC19 expression is positively correlated with follicular helper T cells in most cancers, while CD8+ T cells show no correlation, indicating cancer-type-specific immune modulation. (d, e) ZDHHC19 generally exhibits a positive correlation with chemokine receptors across most cancer types, except for adrenocortical carcinoma, endometrial carcinoma, uterine sarcoma, and uveal melanoma, where it shows a negative correlation. (f) ZDHHC19 shows a significant positive correlation with immune microenvironment scores in various cancers, particularly pancreatic cancer, likely due to its involvement in immune cell chemotaxis.

Lastly, we examined the role of ZDHHC19 in pan-cancer. Box plots and paired box plots showed that ZDHHC19 was significantly upregulated in most cancer types, including bladder cancer and breast cancer, indicating that ZDHHC19 acts as a common oncogene (Figure 8a,b). ZDHHC19 exhibited inconsistent correlations with immune cells across different cancer types. For example, follicular helper T cells were positively correlated with ZDHHC19 expression in most cancers, while CD8+ T cells showed a marked lack of correlation, suggesting that ZDHHC19 requires personalized consideration in guiding immune responses across various cancers (Figure 8c). Regarding chemokine receptors, ZDHHC19 displayed a generally positive correlation with chemokine receptors in most cancers, except for adrenocortical carcinoma, endometrial carcinoma, uterine sarcoma, and uveal melanoma, where it was negatively correlated with chemokine receptors (Figure 8d,e). Furthermore, ZDHHC19 showed a significant positive correlation with immune microenvironment scores in many cancers, especially pancreatic cancer, likely due to its involvement in immune cell chemotaxis (Figure 8f).

## 4. Discussion

This study explores the expression and functional significance of ZDHHC19 in sepsis, focusing on its involvement in immune cell activation, inflammatory pathways, and its potential as a diagnostic and prognostic biomarker. Our findings demonstrate that ZDHHC19 is significantly upregulated in sepsis, particularly in neutrophils, and that its expression correlates with key inflammatory responses and immune cell communication. These results suggest that ZDHHC19 plays a crucial role in immune dysregulation observed in sepsis and may serve as a novel target for therapeutic interventions. In this discussion, we compare our findings with previous studies and explore the broader context of ZDHHC19's role in sepsis and its potential therapeutic implications.

ZDHHC19, a palmitoyltransferase enzyme, plays a critical role in modulating immune cell function by regulating protein palmitoylation. This posttranslational modification influences various cellular processes, such as protein trafficking, membrane localization, and signal transduction, which are vital for immune cell activation and inflammatory responses. Our results are consistent with previous studies indicating that palmitoylation is a potential regulator of immune cell function in inflammatory conditions, including sepsis. In our study, we found that ZDHHC19 expression was particularly high in neutrophils, which are central to the pathophysiology of sepsis due to their role in the early inflammatory response and host defense. Neutrophils are known to release proinflammatory cytokines and proteases during sepsis, contributing to tissue damage and organ dysfunction [31]. The upregulation of ZDHHC19 in neutrophils suggests that palmitoylation pathways may be critical in regulating neutrophil activation and function during sepsis, in line with findings showing that palmitoylation affects neutrophil behavior.

Our findings also reveal that ZDHHC19 expression was associated with the activation of multiple inflammatory pathways, including collagen metabolism, myeloid cell activation, and cell adhesion, which contribute to the progression of sepsis. These findings align with studies identifying inflammatory pathways, such as collagen remodeling, as critical in sepsis pathogenesis [32, 33]. The activation of these pathways in patients with high ZDHHC19 expression suggests that ZDHHC19 may sustain the inflammatory response, exacerbating tissue damage and promoting the persistence of the disease. This is significant in sepsis, where excessive inflammation can lead to multiorgan failure. Our study suggests that targeting ZDHHC19-mediated signaling pathways could modulate the inflammatory response in sepsis.

One of the most noteworthy findings of this study is the observed association between ZDHHC19 expression and clinical severity in sepsis. We found that ZDHHC19 was upregulated in sepsis shock patients and correlated with poor survival outcomes, suggesting its potential utility as a prognostic indicator. Early identification of high-risk patients remains a critical challenge in sepsis management, and while traditional biomarkers such as procalcitonin (PCT) and C-reactive protein (CRP) are widely used, they have limited predictive value for disease progression and outcomes [34, 35]. In contrast, ZDHHC19 may reflect more specific aspects of immune dysregulation in sepsis. Furthermore, its elevated expression in patients with severe disease and increased mortality aligns with its previously reported roles in other inflammatory conditions, including cancer, where it is involved in immune modulation and disease progression. While these findings are promising, we acknowledge that the clinical relevance of ZDHHC19 as a biomarker requires further prospective validation in independent patient cohorts before it can be considered for translational or therapeutic application.

A notable finding of our study was the high specificity of ZDHHC19 expression in CD177+ neutrophils, a subset of neutrophils involved in the pathogenesis of sepsis. CD177+ neutrophils are considered more activated than their CD177− counterparts and play a critical role in the immune response to infections, including sepsis [36, 37]. The observation that ZDHHC19 was highly expressed in CD177+ neutrophils suggests that this subset may be particularly involved in the inflammatory processes of sepsis. Our results align with studies showing that the activation of neutrophils, particularly CD177+ neutrophils, is crucial for sepsis development and its complications. By regulating the activation of CD177+ neutrophils, ZDHHC19 may influence the severity of the immune response during sepsis and contribute to disease progression.

Furthermore, our study showed that ZDHHC19-positive neutrophils had the highest communication with macrophages, another key immune cell type in sepsis. Macrophages are involved in both the initiation and resolution of inflammation, and their dysregulation can contribute to sepsis pathogenesis. The communication between ZDHHC19-positive neutrophils and macrophages, particularly through the RETN-CAP1 receptor-ligand pair, suggests that ZDHHC19 may modulate interactions between these two cell types, amplifying the inflammatory response. This finding aligns with studies demonstrating that neutrophil–macrophage interactions are essential for regulating inflammation and immune responses in sepsis.

Interestingly, our study also revealed that ZDHHC19 is upregulated in various cancer types, including bladder and breast cancer, which aligns with previous research demonstrating its role in tumor progression and modulation of the immune microenvironment. Notably, ZDHHC19 has been implicated in promoting immune evasion in cancer by influencing processes such as antigen presentation suppression and Treg recruitment [28]. These mechanisms bear striking similarity to the immune dysregulation observed in sepsis, where our data show that high ZDHHC19 expression is associated with reduced MHC class II molecule expression, impaired antigen presentation, and a shift toward an immunosuppressive state. These parallels suggest that ZDHHC19 may participate in a shared immune-modulatory pathway that contributes to disease progression across distinct pathological contexts. Understanding how ZDHHC19 alters immune responses in both cancer and sepsis may offer new avenues for developing immunomodulatory therapies aimed at restoring immune balance and improving clinical outcomes.

This study has several limitations. First, the use of heterogeneous publicly available datasets from different platforms and clinical settings may introduce batch effects and sampling bias, despite our efforts to correct for technical variability using integration algorithms like Harmony. Second, our findings are based entirely on computational analyses, which, while informative, rely on algorithmic assumptions and cannot fully capture the complexity of biological systems. As such, the associations identified—particularly the role of ZDHHC19 in immune modulation and prognosis—should be considered exploratory. Finally, the absence of experimental validation limits our ability to confirm causality. Future studies involving functional assays and in vivo models are necessary to substantiate these findings and assess the therapeutic potential of targeting ZDHHC19 in sepsis.

## 5. Conclusion

In conclusion, our study provides strong evidence that ZDHHC19 plays a significant role in the immune response during sepsis, particularly in neutrophils, and may serve as a novel diagnostic and prognostic biomarker for the disease. The upregulation of ZDHHC19 in sepsis shock patients and its correlation with poor survival outcomes highlight its potential as a marker of disease severity. Furthermore, the high specificity of ZDHHC19 expression in CD177+ neutrophils suggests this enzyme may play a critical role in neutrophil activation and inflammation during sepsis. Our findings are consistent with previous research on immune cell activation and inflammation in sepsis pathogenesis, offering new insights into the molecular mechanisms underlying the disease.

## Data Availability

The data that support the findings of this study are available from the corresponding authors upon reasonable request.
